# Evaluation of a technology-enhanced, integrated community health and wellness program for seniors (HWePS): protocol of a non-randomized comparison trial

**DOI:** 10.1186/s12889-022-14921-z

**Published:** 2023-01-05

**Authors:** Hongsoo Kim, Hyoungshim Choi, Young-il Jung, Eunji Kim, Woojoo Lee, Jae Yoon Yi

**Affiliations:** 1 Department of Public Health Science, Graduate School of Public Health, Seoul National University, 1 Gwanak-Ro, Gwanak-Gu, Seoul, South Korea; 2 Institute of Health and Environment, Seoul National University, 1 Gwanak-Ro, Gwanak-Gu, Seoul, South Korea; 3 Seoul National University Institute of Aging, 1 Gwanak-Ro, Gwanak-Gu, Seoul, South Korea; 4 College of Nursing, Hansei University, 30 Hansei-Ro, Gunpo-Shi, Gyeonggi-Do, South Korea; 5 Department of Environmental Health, National Open University, 86 Daehak-Ro, Jongno-Gu, Seoul, South Korea

**Keywords:** Health equity, Integrated community care model, Personalized care, Health literacy, Digital technologies, Geriatric care model

## Abstract

**Background:**

Healthy aging for all in the community is a shared public health agenda for countries with aging populations, but there is a lack of empirical evidence on community-wide preventive models that promote the health of older people residing in socially-disadvantaged communities. The Health and Wellness Program for Seniors (HWePS) is a technology-enhanced, multi-level, integrated health equity intervention model. This study evaluates the effect of the HWePS on the health and well-being of older adults residing in urban, low-income communities.

**Methods/design:**

HWePS is a prospective, non-randomized comparison trial conducted in an intervention and a control neighborhood (*dong*) in Seoul, South Korea, over 12 months. Older people who reside in the small areas and meet the inclusion/exclusion criteria are eligible to participate. The multi-level, multi-faceted HWePS intervention is a preventive community care model for older residents guided by the expanded chronic care model, the comprehensive health literacy intervention model, and the Systems for Person-centered Elder Care model along with health equity frameworks. HWePS consists of four components: a health literacy intervention based on individual and community needs assessments, personalized (self-)care management featuring nurse coaching and peer support, a healthy-living and healthy-aging community initiative, and information and communication technology (ICT) systems. The primary outcomes are self-reported health and health-related quality of life. Outcome assessors and data analysts are blinded to group assignment. Process evaluation will be also conducted.

**Discussion:**

As a multi-level health equity project, HWePS has adopted a novel study design that simultaneously targets individual- and community-level factors known to contribute to health inequality in later life in the community. The study will provide insights into the effectiveness and implementation process of an integrated, multi-level, preventive community care model, which in turn can help improve the health outcomes of older residents and reduce disparities in underserved urban communities.

**Trial registration:**

ISRCTN29103760. Registered 2 September 2021, https://www.isrctn.com/ISRCTN29103760

## Background

Healthy aging is a widely accepted common goal of most countries with aging populations, regardless of income level. The proportion of older adults in the world’s population is expected to reach 16% by 2050 [1] and a majority of these older people are also expected to reside in urban communities [2]. Various policy efforts to promote healthy aging have been made at the country and international levels [3, 4]. Often overlooked, however, is that opportunities for healthy aging are unequally distributed within and between countries [3]. Another gap in the healthy aging intervention literature is that most interventions target health behaviors at the individual level only [5, 6], despite the potential impacts of the communities where the individuals reside. Yet another gap is that healthy aging interventions and programs often target those with specific diseases, or risks, for whom the intervention effects can be clearer; the generalizability of those studies is limited [7]. Together, these gaps have resulted in a lack of evidence on community-wide preventive models promoting healthy aging for relatively healthy community-dwelling older adults and, in particular, those who are socially disadvantaged.

Health equity is a critical goal of public health policy and practice. Beyond mere surveillance and monitoring of health disparities, heath equity interventions aiming to reduce and/or eliminate health disparities have been designed and tested [8, 9]. However, these interventions often fall short of achieving health equity, partly attributable to gaps in knowledge and translation [10]. Interventions are often focused on downstream—rather than upstream—social determinants of health [11, 12]. A lack of understanding of the pathways through which social factors influence health is another critical gap. Multi-level, population-tailored approaches along with community engagement have been key recommendations for disparity studies in public health and intervention science [10, 13].

To address such gaps in the healthy aging and health disparity literature, public health interventions promoting health equity for community-dwelling older adults are needed. This study aims to develop and evaluate an integrated health equity intervention promoting the health and wellness of older people in an urban, low-income community in Seoul, South Korea (henceforth *Korea*). Korea is an East Asian country experiencing the most rapid population aging and the highest elderly poverty rate among the OECD countries [14]. As a consequence of rapid urbanization over the last 30–40 years, there is a large number of urban, lower-income elderly, and meeting their health and care needs is essential to promoting healthy aging for all. Seoul, the capital of Korea, is a mega-city with a population of 9.9 million in 2022, among which about 17.2% are older adults and about 350,000 are older adults living alone [15]. The city health department places public health nurses in each of the approximately 420 community service centers in the city [16], a bold step to increase access to preventive public health services for the poor and/or older residents in local communities and a move that has now been benchmarked by many other provinces in Korea. Yet unwarranted variations still exist in health care access and outcomes across communities in Seoul and across the country, and such gaps have not been easily narrowed [17]. Under the COVID-19 pandemic, it is also essential to improve access to care and to protect the health and well-being of older people in low-income communities, where infection rates and the negative impacts of service closures related to social distancing measures are higher compared to their counterparts [18].

### Theoretical rationale

The Health and Wellness Program for Seniors (HWePS) is a technology-enhanced, multi-level, integrated health equity intervention model that aims to promote the health and wellness of older people in an urban, low-income community. As a small-area, public-health equity program (intervention), the HWePS was designed based on several theoretical and conceptual models. The components and process of the HWePS intervention are guided by the expanded chronic care model (ECCM) [19], the Systems for Person-centered Elder Care (SPEC) model [20], and the comprehensive health literacy (HL) intervention model [21] as well as the health inequality models described below. Guided by the Commission on Social Determinants of Health (CSDH) conceptual framework [22], the intervention assumes exposure and vulnerability to health-comprising conditions vary by social status, a structural determinant of heath (SDH) inequalities; it also hypothesizes that a health system is not an SDH in itself but rather an important mediator that can change the health and wellness of people through access to care and intersectoral action.

The Bay Area Regional Health Inequities Initiative’s (BARHII’s) public health framework for reducing health inequalities is a decision-making framework for the California Department of Public Health [23]. In order to address heath inequalities, the conceptual framework emphasizes the focus of public health practices should move toward living conditions as well as institutional and social inequalities (middle and upstream determinants), beyond the traditional medical model-based practice mainly focusing on individual risk behaviors and disease and injury (downstream determinants). Underpinned by the emerging public health practice paradigm in BARHII’s framework, the HWePS is designed to improve living conditions, in particular the health and social service environment, by creating a healthy living and healthy aging environment at the neighborhood (small-area) level. The living environment, according to BARHII’s framework, has a critical role as a modifier to reduce the impacts of upstream factors of inequalities on individual health. A healthy-living, healthy-aging initiative including a wide range of activities for community capacity-building and civil engagement is a critical component of the HWePS intervention. The activities were mainly selected based on suggestions and requests from older residents and community leaders in collaboration with public health officials, community service centers, and NGOs, with available resources and programs in the Jungnang-gu (district) and also, more broadly, the city of Seoul.

Along with this community-level healthy aging initiative, the HWePS addresses determinants of health inequality at the individual and interpersonal (social) levels, which assumes each older adult has unique needs and preferences in developing their optimal healthy aging trajectory [3]. A personalized care-management program featuring care planning and coaching that supports each older person in maximizing knowledge, skills, and competency for self-care, is essential for improving health and well-being in later life.

The individual-level intervention components of the HWePS were designed by adopting and modifying the components and process of the Systems for Person-centered Elder Care (SPEC) [20, 24, 25]. SPEC is an integrated geriatric care model, developed and tested at nursing homes in Korea, that significantly improved quality of care for frail older adults with multiple chronic illness [20]. The geriatric assessment-based care planning in the SPEC is also a vital component of the individual-level intervention in the HWePS. The participants and scope of the SPEC’s interdisciplinary case-conferences and care-coordination have been expanded for the community-based, civil-engaged HWePS intervention. ICT tools, another component of the SPEC model, are also a core component of the HWePS model. The expanded chronic care model [19], which emphasizes the importance of community capacity-building for health promotion beyond traditional health systems and institutions, not only guided the further development and tailoring of the SPEC intervention model developed under the chronic care model [26] but also is the theoretical framework of the community-based HWePS.

Lastly, the HWePS was guided by the comprehensive health literacy (HL) intervention model emphasizing the critical role of health literacy in reducing heath inequalities and promoting healthy aging [21]. The HL model suggests interventions should target the following five factors [21]: the context of the individual, via interventions strengthening community social support (e.g., family, peers, communities); the individual, via interventions improving each person’s low heath literacy (e.g., person-centered capacity-building and self-management); the interaction between the individual and the heath system, via interventions to improve communication between individuals and health professionals; health professionals, via interventions that aim to improve their literacy capacities; and the communication within and accessibility of health systems, via interventions that aim at reducing barriers to access or policies to improve care quality. Guided by the model, we designed a health literacy intervention to be a critical component of the HWePS, comprising a community-wide, multi-channel health information delivery service and individualized self-checkups evaluating a participant’s intrinsic capacity using an evidence-based clinical decision support tool based on the interRAI Check-Up for Self-Report (CUSR) [27].

During the COVID-19 pandemic, delivering valid and reliable information tailored to the community and population’s needs can help promote heath literacy and better communication with the health system [28, 29]. We integrated the five strategies of the comprehensive HL intervention model [21] into the HWePS intervention, facilitating various interactions and communication activities between older people, their families, community lay health leaders (CLHLs; peers), and nurse coaches and other multidisciplinary team members (heath and social professionals). We aim to increase the health literacy of various intervention participants (older people, peers, and health/social professionals) in the low-income urban community as a whole.

## Objectives

Improving health equity in later life should be a top priority of public health officials, especially considering the possibility of extended later life due to increasing longevity and the proportion of older age groups. In order to advance healthy aging for all, it is imperative to reduce health disparities among older people residing in urban, low-income communities in Korea, for which effective community-based health promotion strategies considering social determinants of health are needed. Given the context, this study aims to evaluate whether or not the HWePS, a community-based, multi-level, preventive and integrated elder care model will improve the health and quality of life of older residents in an urban, low-income community, compared to usual-plus care. The HWePS is well-aligned with a nationwide policy goal of building community-based integrated care systems for older people and promoting an aging-in-place policy [30].

## Methods/design

### Study design

**Fig. 1:**
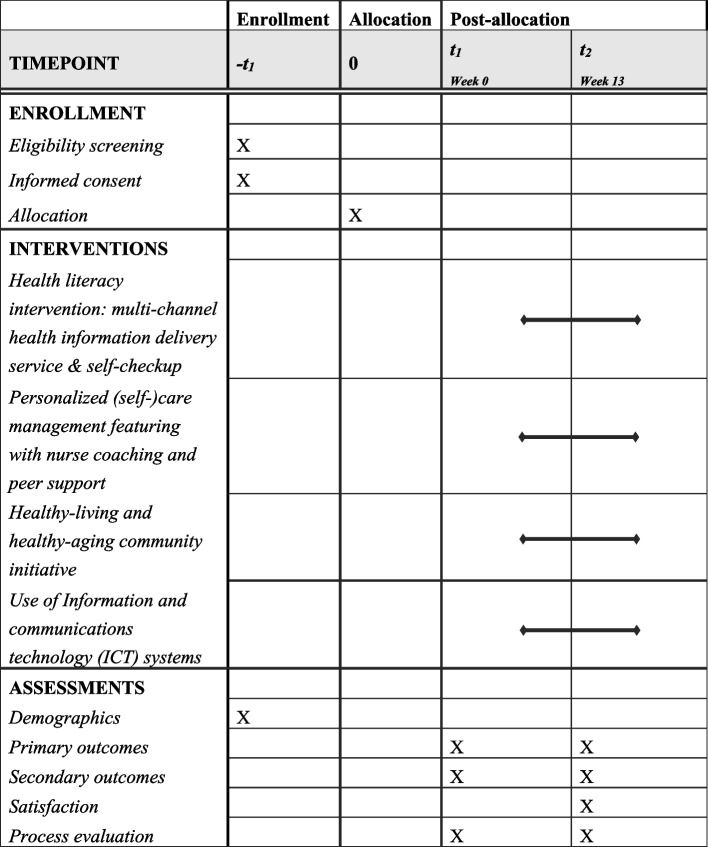
The schedule of enrollment, interventions, and assessments of the HWePS program

The Health and Wellness Program for Seniors (HWePS) is a two-arm, non-randomized, controlled cluster trial that seeks to evaluate a theory-guided, technology-enhanced, integrated health-equity intervention model for older residents in urban, low-income communities in Seoul, Korea, during the COVID-19 pandemic. The HWePS targets complex, multi-level factors influencing disparities, including factors at the individual, interpersonal (social), and community levels [10]. The schedule of enrollment, intervention, and assessment of the intervention is summarized in Fig. 1. A total of two measurements (pre- and post-intervention) are conducted on each individual. A 12-week case management program aiming to build self-care skills and practice with a nurse coach and peer support is rolled out in three clusters and spans an overall study period of approximately 12 months. A community-wide, multi-channel information delivery service aiming to promote heath literacy, and a healthy-living, healthy-aging initiative aiming to build community capacity and resources/networks are continued throughout the entire intervention period. This intervention was developed through a mixed-methods approach guided by 6SQuID [31]; various stakeholders have been involved in developing the heath disparity intervention project, starting with its inception and continuing throughout the entire intervention development process, which will be published as a separate paper. The trial described here is prospectively registered at ISRCTN. This protocol has been reported following TREND guidelines [32].

### Study setting and participants

The HWePS is being conducted in two urban neighborhoods (*dong*; 1 intervention *dong*, 1 control *dong*) within Jungnang-gu in Seoul, Korea. Jungnang-gu, one of the easternmost districts of Seoul, is home to a higher-than-average proportion of older adults living alone (8.7% vs. 7.1% in Seoul) and people receiving national basic livelihood security (7.0% vs. 4.1% in Seoul). Out of the 25 districts in Seoul, Jungnang-gu has the third lowest fiscal independence rate as of 2020 [33]. The intervention and control areas (*dong*) within Jungnang-gu have similar proportions of older residents, but they are geographically remote from each other in order to prevent any spillover effect. The selection of these particular neighborhoods was also based on health statistics about the areas as well as input from the public health center in Jungnang-gu regarding service needs in the intervention areas.

Study participants include older adults residing in the intervention and control areas who consent to participate in the research study. Participants come from a pool of individuals who participated in a community-wide community health and wellness survey conducted in late 2020 [34]. Inclusion criteria for participants are as follows: 1) aged 65 or older, 2) non-frail based on the K-FRAIL [35], 3) not a long-term care insurance beneficiary, and 4) not planning to move away from the area during the intervention period. Study participation is entirely voluntary, and participants may withdraw from the study at any time.

### Intervention

#### Components of the HWePS

1. Health literacy intervention based on individual- and community-needs assessment

The HWePS aims to increase personal and also community health literacy through a community-wide, multi-channel health information delivery service and individualized self-checkups of functional capacity. The former aims to increase the access of community residents to valid and reliable heath information tailored for the information needs of the community during COVID-19, when community health and social services are closed or largely reduced. The contents of the health information service have been curated based on a needs assessment via a community-wide health and wellness survey in late 2020 [34] and other relevant reports/statistics, as well as various input obtained during the intervention development process from residents, community leaders, clinical professionals, and health and administrative officers in the community. The evidence-based contents cover a wide range of health information aiming to prevent frailty and promote healthy aging, including suggestions for improving physical activity, nutrition/diet, mental health, social relationships, and safety measures related to COVID-19. This health information is delivered regularly via SMS as an e-poster or via postal mail according to each older person’s preference. The information delivery service is augmented by weekly in-person or phone counseling provided by community nurses, an optional service for anyone who would like to ask questions for a better understanding of the delivered information and/or seek further information and services.

The self-checkup is a decision-support service that aims to help older persons assess and be aware of their own strength, weakness, and risk factors in maintaining and promoting functional capability [3]. The self-checkup service is a smartphone application-based, multi-dimensional functional assessment tool using the interRAI Check-Up Self-Version (CUSR) and other intervention-relevant items. The CUSR is a valid and reliable self-assessment tool designed to examine the health and functional status of relatively healthy community-dwelling residents by themselves or with the assistance of family members/lay helpers [27, 36]. Upon completion of a 90-item health check-up, the CUSR generates a report including key function scales and a list of clinical assessment points for common geriatric health risks (e.g., pain, falls, inactivity, smoking/drinking, nutrition, cardiovascular disease, etc.) using triggers derived from evidence-based algorithms. The self-checkup experience is expected to motivate older persons to seek appropriate health information and services relevant to their own health risks, empower them to make a self-care plan, become interested in self-care skill-building activities, and practice a healthy lifestyle, all which are the contents of the (self-)care management component of the HWePS described below.

2. Personalized care management featuring nurse coaching and peer support

The personalized care management grounded on self-checkup results is based on the SPEC intervention model [20, 25]. It features care planning with a nurse coach and self-care skill-building with peer support. Care planning with a nurse coach is a critical component of the HWePS to empower each older person to develop their own action plan to build their self-care capacity reflecting their own preferences, choices, and timeline for health promotion goals, action plans, and activities. Older participants execute daily self-directed care plans with the guidance and support of a nurse-led multidisciplinary care team along with trained community lay health leaders (CLHLs). The multidisciplinary care team consists of a lead nurse, nurse coaches, a fitness specialist, a nutritionist, and a social worker. The nurse coaches are community health nurses (CHNs) for the “visiting community service center service” program in the intervention neighborhood who are trained for this role; in addition, a CHN will take on the role of lead nurse during the dissemination phase of the intervention. Based on the results of the individualized needs assessment, the nurse coach follows evidence-based protocols to guide older adults’ care plans and guides CLHLs to provide peer support assisting older adults to reach desired goals and outcomes.

The care management program is a 12-week, self-care skill-building, multidisciplinary, tailored intervention with peer support from trained CLHLs. At the beginning of the care management intervention, a self-checkup using the CUSR is conducted by each older participant with the support of a trained CLHL (a peer in the community) through an interview; the CLHL assists the older person to read the CUSR items and/or fill out the electronic questionnaire. Upon completion of the CUSR, the CLHL reviews the automated report and assessment results with the participant and delivers 1- to 2-page educational pamphlets describing each of the identified health risks derived from a total list of common geriatric health issues. The care management process involves the assignment of a nurse coach; reviews of older adults’ health needs and preferences as determined by the CUSR and social and literacy items from the community health and wellness survey; goal setting; personalized health information; and delivery of tailored exercise, nutrition, mood, and social intervention programs, along with a 12-week program promoting walking and other physical activities. Using the HWePS app, older adults can set their own tasks toward achieving their health goals, and CLHLs provide peer support to empower and strengthen older people’s self-care capacity using the app’s to-do checklist set by the older adult. In addition, multidisciplinary care conferences (MCCs) are held for more in-depth team discussions on care planning and coordinated delivery for complex or especially vulnerable older adults. During an MCC, the initial care plan is refined based on in-depth team discussion. If a referral to community resources such as medical or public welfare services is required, a request form is prepared and sent to the relevant institutions.

3. A healthy-living and healthy-aging community initiative

The importance of addressing upstream social determinants of health has been emphasized for reducing health inequity [37]. Guided by the BARHII and the ECCM models [19, 23], the HWePS goes beyond individual-level care planning to develop community-wide healthy living conditions promoting healthy aging. Various community participatory activities (CPAs) exist under the community-wide initiative, aiming for 1) creating a supportive environment, 2) strengthening community capacity, and 3) building community resources/networks [19]. The CPAs, which seek to create a supportive living environment in which older adults can pursue a healthy life, include services and programs such as age-friendly smartphone subscriptions, smartphone education, and home installation of safety bars for high-risk groups. “Strengthening community capacity” is a component that develops the community’s health-oriented capabilities to utilize local resources and enables community members to identify and solve local health problems on their own. The CPAs include community health-education sessions and training CLHLs as health activists in the community. The CLHLs include people over 50 years of age residing in the intervention area and serve as key players in delivering the HWePS model within the community. Lastly, building community resources/networks involves organically connecting the various health care and welfare services that are segmented within the community and making referrals to participants as needed. Through regular meetings, research teams collaborate with various stakeholders to continuously identify local needs, search for and find relevant human and material resources, build strategic partnerships within the region to develop a myriad of community-level resources and networks, and develop and implement new services and programs together.

These CPAs under the healthy-living, healthy-aging community initiative aim to address older adults’ social determinants of health by building a foundation to empower the community’s health efficacy. Participating stakeholders include the researchers and the multidisciplinary intervention team as well as local stakeholders including older residents, community leaders, health and social professionals, civic groups, and the advisory committee. Monthly meetings are held with local public health stakeholders to identify existing community resources to which referrals may be made and to solidify new partnerships and integrate them into the community. The newly developed community-level healthy aging programs and services are managed by the senior health and wellness center, and individual referrals are made by the multidisciplinary care team members as required.

4. Information and communications technology (ICT) systems

HWePS includes a multi-channel health information service for older participants that mainly delivers information as an e-poster via KakaoTalk, a multifaceted, free SMS application. For those without a smartphone or who lack the relevant digital literacy, printed information packets are delivered by postal mail. For personalized care management, a prototype HWePS information system has been developed for research purposes, including a smartphone application (hereafter the HWePS app) for end users (seniors, lay leaders, health professionals) and a cloud-based website for managers (health professionals, the research team). The HWePS information system includes modules for self-checkups and needs assessments; care planning and monitoring; education; and community resources for older adults, lay leaders, and/or health professionals. These ICT tools facilitate online communication between seniors, lay leaders, professionals, and the research team, which is especially helpful for implementing the HWePS program during COVID-19. Furthermore, the cloud-based system allows for real-time access to collected data, facilitating communication between members of the care team and the research team. Throughout the intervention, the research team also actively utilizes KakaoTalk for various purposes including progress checks on intervention delivery and/or data collection and reminders. Further details on all the HWePS intervention components are summarized in Table 1.

**Table 1:** Description of the HWePS intervention: components, theoretical rationale, aims, and implementation information

Component	Theoretical Rationale	Detailed Intervention Description	Specific Aim	Intervention Level
1. Health literacy intervention based on both community and individual needs assessments	- ECCM: Decision support - Comprehensive HL intervention model	- Community-wide, multi-channel health information delivery service (SMS or postal mail) based on a community health survey and other relevant reports/documents related to community health needs - Individualized needs assessment using interRAI CUSR producing health and functional profiles and risk triggers based on evidence-based algorithms	- Promote health literacy for older people, community lay health leaders (CLHLs), and the care team - Increase health awareness - Facilitate communication between the community and the public health center (the intervention team)	-Individual -CLHLs (peers) & health professionals - Community
2. Personalized (self-)care management with a nurse-led multidisciplinary team and peer support by community lay health leaders (CLHLs)	- ECCM: Self-management; developing personal skills; delivery system design - SPEC intervention model	- Self-care planning by older people with a nurse coach, based on the CUSR report - Features nurse coaching for self-care goal setting and skill-building education/planning, and peer support (by trained CLHLs) for keeping to the plan - Tailored intervention and information (SMS) programs for one or more triggered/preferred healthy life-style areas (diet, exercise, mood, and social relationship) by multidisciplinary care team - Community-wide walking promotion program - Nurse-led multidisciplinary care conferences (MCCs) attended by both professionals and CLHLs	- Enhance self-care of identified health problems - Improve health behaviors through self-care and coaching from care team and CLHLs - Improve health outcomes and efficacy through self-care and walking promotion - Increase easily accessible resources within the community as a window for health needs and referrals - Strengthen social support in the community	Individual
3. Healthy-living and healthy-aging community initiative	- ECCM: Creating supportive environments; strengthening community capacity; building community resources, networks & partners - BARHII Framework	- Activities for creating supportive environment (e.g., smartphone subscription program, smartphone education, safety-bar installation for high-risk households) - Activities for strengthening community capacity (e.g., community health education sessions, CLHL training) *-* Activities for building community resources/networks and community partners^a^ (e.g., referrals to community center nurse, social welfare center, medical center)	- Improve living conditions for healthy aging, in particular, health and social care service conditions - Address HRSNs and social determinants of health	Community
4. HWePS information and communications technology (ICT) system	- ECCM: Information systems - SPEC intervention model	- Use of the HWePS project smartphone application (the HWePS app, for research purposes) and a cloud-based website for care management - Use of KakaoTalk for communication between research team, multidisciplinary care team, CLHLs, lay assessors	- Promote effective and efficient information exchange and communication between players - Collect and store electronic data in a secured information system	-Individual - CLHLs (peers) & health professional - Community

*ECCM* The extended chronic care model [19], *HL* Health literacy model [21], *SPEC* The System for Person-centered Elderly Care [25], *BARHII* The Bay Area Regional Health Inequities Initiative [23], *HRSN* Health-related social needs, *CLHL* Community lay health leaders, *SMS* Short message service, *MCC* Multidisciplinary care conference

^a^Community partners include local health and social care providers, NGOs, public health centers, community service centers, Jungnang district office, the health promotion department of the City of Seoul, etc.

In summary, participants in the intervention area will receive the entirety of the HWePS intervention comprising a health literacy intervention including a community-wide, multichannel, health information delivery service and individualized self-checkups; a 12- week, technology-enhanced care management program for care planning and self-care skill-building through nurse coaching and peer support; and a community-wide initiative promoting healthy aging and aiming in particular to address living conditions and health/social care service conditions, midstream/upstream causes of health inequality, by integrating a wide range of services meeting health and health-related social needs. All services are arranged and based in a senior health-wellness center, the basecamp for the intervention team, located at a five-minute walk from the community service center in the intervention area. The wellness center also coordinates delivery of small prizes (e.g. food packages for nutrition, exercise equipment, personal hygiene amenities, etc.) for older adults in the tailored programs to encourage ongoing participation throughout the intervention period.

During the entire intervention period, the participants in the control area will receive a community-wide health information delivery service, which is a part of the literacy intervention. Delivery of the health information will be carried out identically to the intervention group through KakaoTalk (an SMS program widely used in Korea), or postal mail. Other than receiving health information, the control region will not receive any other components of the intervention, such as individualized needs assessments through self-checkups using the CUSR, tailored care planning, or community-level healthy aging capacity-building and empowerment.

### Outcomes

The study seeks to evaluate two primary outcomes: self-rated health and health-related quality of life (HRQoL), along with several secondary outcomes and process evaluation measures. Most primary and secondary outcomes are taken from the annual Community Health Survey, a nationwide survey conducted by the Korean CDC [38]; outcomes from other sources are indicated accordingly. All outcomes and their measurements are summarized in Table 2.

**Table 2:** Overview of the outcome variables, measures, and observation points

Outcome	Variable	Data Source/Instrument	Measurement Points	Target Respondents
Primary	Self-rated health	WHO World Health Survey; Korea Community Health Survey	T1, T2	IG, CG
	Health-related quality of life	EuroQol (EQ)-5D-3L	T1, T2	IG, CG
Secondary	Frailty	Fried’s frailty phenotype	T1, T2	IG, CG
	Wellbeing	World Health Organization- Five Well-Being Index (WHO-5)	T1, T2	IG, CG
	Self-efficacy	Korean Self Rated Abilities for Health Practices (K-SRAHP)	T1, T2	IG, CG
	Community efficacy	Collective Efficacy Scale	T1, T2	IG, CG
	Physical activity	Korean version of the International Physical Activity Questionnaire (K-IPAQ) as presented in the Korea Community Health Survey	T1, T2	IG, CG
	Hypertension awareness, diabetes awareness, mood, stress	Single items taken from the Korea Community Health Survey	T1, T2	IG, CG
	Functional health	Scales and CAPs from interRAI Check-Up Self-Rated Version (CUSR)	T1, T2	IG
	Technology acceptance	Short version, Senior Technology Acceptance Measure (STAM)	T1, T2	IG
	Nutrition, depression	Nutrition Questionnaire Elderly (NQE), Patient Health Questionnaire-9 (PHQ-9)	T1, T2	IG participants who received tailored programs
	Satisfaction	Client Satisfaction Questionnaire	T2	IG

*IG* Intervention group, *CG* Control group

#### Primary outcome measure

*Self-rated health* is measured with a single item that asks, “In general, how do you consider your overall health?” using a 5-point scale (1 = Very good; 5 = Very poor). Self-rated health is a widely-used measure of subjective morbidity [39], which has been validated in various settings including for health-equity analyses [40]. *HRQoL* is measured using the Korean version of the EuroQol-5 Dimensions, three-level version (EQ-5D-3L), a widely-used standardized measure of health-related quality of life with good reliability and validity across international samples [41]. The EQ-5D-3L assesses five dimensions of health (mobility, self-care, usual activity, pain/discomfort, and depression/anxiety), and each domain is rated from 1 to 3, with 1 meaning no problems, 2 meaning some problems, and 3 meaning extreme problems.

The primary outcomes will be measured at two time points: at T1 (Week 0; upon enrollment) and T2 (Week 13; following the end of the 12-week personalized intervention period).

#### Secondary outcome measures

*Frailty* will be measured using Fried’s frailty phenotype [42], composed of five domains: weakness, slowness, exhaustion, low physical activity, and unintentional weight loss. Weakness and slowness are performance-based items, assessed by grip strength and gait speed respectively, adjusted for gender and body mass index/height. Grip strength is measured using the CAMRY EH-101 electronic hand dynamometer, and gait speed is measured using a tape measure of 2.5 m. Exhaustion, low physical activity, and weight loss are derived from survey-based self-report items. Each component is scored 0 or 1 with a final score of 0 (healthy) to 5 (frail).

*Wellbeing* is measured using the WHO-5 Well-Being Index (WHO-5), a short questionnaire that assesses the subjective well-being of patients. Each item is rated from 5 (always) to 0 (never), and the sum score of all items is utilized. The WHO-5 has been translated into Korean with acceptable reliability (0.83) [43].

*Self-efficacy* will be measured using the Korean Self-Rated Abilities for Health Practices (K-SRAHP) health self-efficacy measure [44]. The measure, based on Becker et al.’s (1993) health self-efficacy scale [45], has been revised and tested for validation in a Korean sample [44]. A total of 24 items of health self-efficacy covering 6 domains (physical activity, disease management, emotional regulation, nutrition, stress management, and health behavior) are measured and rated on a 5-point scale from 0 (not at all) to 4 (completely). A composite score of 0 to 96 is utilized for the analysis.

*Community efficacy* of the residential area is measured using five items that have been revised based on Sampson et al.’s (1997) Collective Efficacy Scale [46] to better reflect the Korean situation [47]. Collective efficacy is measured by two sub-concepts, informal social control and social cohesion/trust. The five items are measured on a 5-point scale ranging from 1 (strongly agree) to 5 (strongly disagree) and reverse-coded so that larger values indicate greater collective efficacy. The reliability of the five items has a Cronbach’s alpha of 0.681 [47].

*Physical activity* is measured using the Korean version of the International Physical Activity Questionnaire (IPAQ) short form [48]. The IPAQ is a self-reported measure that assesses the amount of physical activity (vigorous, moderate, walking, sitting) performed over the past 7 days. The IPAQ is used to compute respondents’ *moderate to high exercise rate* (more than 20 min of vigorous physical activity, 3 or more days a week OR more than 30 min of moderate physical activity, 5 or more days a week) and *walking practice rate* (more than 30 min of walking, 5 or more days a week), using algorithms from the Korean Community Health Survey [38].

*Hypertension awareness*, *diabetes awareness, mood,* and *stress* are all secondary outcomes measured by single items from the Korean Community Health Survey [38]. *Hypertension awareness* and *diabetes awareness* seek to assess whether the respondent is aware of their blood pressure and sugar levels, both indicators that can influence healthy lifestyles [49]. *Mood* is assessed by the yes/no question, “In the past 1 year, have you experienced sadness or hopelessness that interferes with your daily life for two or more consecutive weeks?” *Stress* is measured by 4-point scale in response to a question that asks how much stress the respondent experiences in his/her daily life (1: very much – 4: not at all). The item is dichotomized into experiencing stress [1, 2] and not experiencing stress [3, 4].

*Technology acceptance* is measured with the short version of the Senior Technology Acceptance Measure (STAM), which was developed based on the Technology Acceptance Model [50]. The STAM is a 14-item, self-rated questionnaire using a 10-point Likert scale, and it consists of 4 domains as follows: attitudinal beliefs, control beliefs, gerontechnology anxiety, and health. There was no existing STAM questionnaire in Korean, so with the approval of the instrument developer group [50], a Korean version of the STAM was developed through forward- and back-translation guided by the process of translation and adaptation of research instruments of Chen et al.

*Functional health* is assessed from the scales and clinical action points (CAPs; e.g., falls, cardiovascular disease, pain, etc.) in the interRAI Check-Up Self-Report (CUSR) [27]. The interRAI CUSR is a multidomain geriatric assessment to screen older adults’ intrinsic capacity, designed to be administered by themselves and/or with assistance from non-health professionals in the community or primary care setting. The CUSR has good psychometric properties in other countries [27, 36] and also in Korea [34].

*Nutrition* and *depression* are measured for those who receive tailored intensive intervention programs (nutrition, emotional). For those who participate in the intensive nutrition program, the Nutrition Quotient for Elderly (NQ-E) is used to measure the nutritional status and dietary quality of older adults [51]. The NQ-E assesses nutritional status through 19 items across four domains (balance, variety, restriction, dietary behavior), which are weighted and summed to determine the respondent’s nutrition rating: low, medium–low, medium–high, or high. For those who participate in the intensive emotional program, the PHQ-9 is used to measure depression. The PHQ-9 comes from the Patient Health Questionnaire developed by Kroenke et al. [52] and translated into Korean by Han et al. [53]. The Korean version of the PHQ-9 has good internal consistency (0.88) and convergent validity (0.74).

*Program satisfaction* is collected using a modified version of the Client Satisfaction Questionnaire, with 8-items that evaluate the participants’ program satisfaction. The measure, which has been translated by Hwang [54], is widely used for Korean public programs.

#### Process measures

**Fig. 2:**
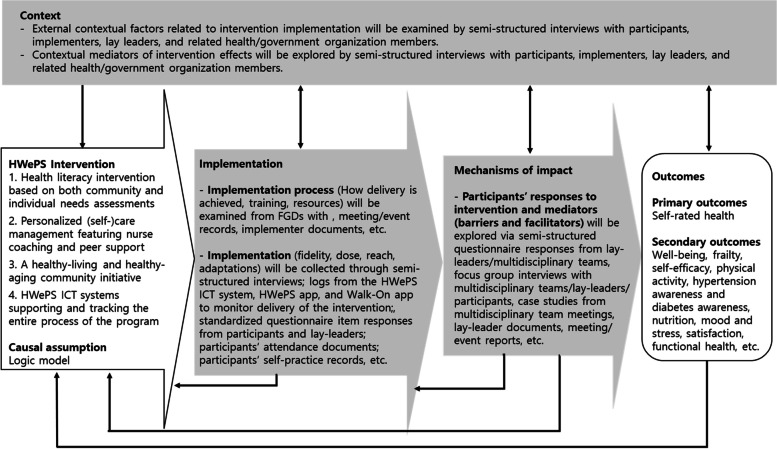
Process evaluation constructs and data collection methods adapted from the UK Medical Research Council (MRC) [56]

The goal of process evaluation in this study is to understand the factors that can influence the success of complex interventions [55]. Guided by the Medical Research Council (MRC) guidance on process evaluation, we developed a framework process evaluation for the HWePS intervention to evaluate the key constructs of context (external factors related to the intervention), implementation process, implementation (fidelity, dose, reach, and adaption), and mechanisms of impact (unintended outcomes and mediating pathways) [56] (Fig. 2). The process evaluation will use a mixed methods design. Quantitative and qualitative data will be collected using standardized questionnaires, case studies, documents, and focus group interviews guided by semi-structured questions, etc.

### Sample size and recruitment

This HWePS intervention study is part of a larger community-wide health equity initiative, starting with a community-wide health and wellness survey conducted from November 2020 to January 2021. The sample for the community-wide survey consisted of approximately 700 older adults in the intervention area and 500 older adults in the control area, which is approximately 19.2% and 7.6% of all older adults in each area, respectively (as of October 2020). Informed consent concerning the entire study was obtained by the research team prior to initiating the survey.

The recruitment of participants into the HWePS intervention is based on the roster of older adults who participated in the community survey. The sample size of the HWePS intervention is calculated using the G*Power 3.1.9.2 program as follows. We hypothesize an effect size (as the input parameter for G*Power) of 0.2 based on previous community-based health and wellness interventions for older adults’ self-rated health [57–59]. The required number of samples is identified as 265 for each arm, by choosing the MANOVA approach in two repeated measures (90% power, 5% α error) in two independent groups. With an estimated dropout rate of 30% in the intervention period, a total sample size of approximately 379 participants per arm needs to be recruited.

Enrollment into the study will progress sequentially by clusters based on residents’ *tong* (smaller neighborhood). At the beginning of each intervention period, all individuals in the cluster will be contacted by the research team by telephone and given a brief explanation of the program. We will directly visit the homes for recruitment of those older adults who do not respond to the phone calls due to low technology literacy, wariness, or other reasons. Upon agreeing to participate in the study, the participant will be scheduled for a health survey for baseline data collection by an external assessor. Recruitment and baseline data collection will be simultaneously conducted. Eligibility and consent to participate is reconfirmed by phone or in-person during the intervention recruitment.

### Assignment of interventions and blinding

Participant assignment into the intervention group or the control group is solely based on area of residence. Participants reside in 15 *tongs* in the intervention area and 15 *tongs* in the control area, and are divided into 3 clusters matched based on mean age and residential type (apartment vs. house).

Considering the nature of the study, limited single-blinding is applied to the intervention, as participants will not be told whether they reside in the intervention or control area, but researchers will be aware of the allocation. Upon giving informed consent, participants in the intervention group will receive an explanation of the entire HWePS intervention, while control group participants will receive an explanation of only the health information component. Assignments will not be explicitly expressed to external assessors but may be inferred throughout the course of the study. An independent data analyst blinded to the assignments will receive a cleaned dataset without any identifiable information.

### Data collection

Data collection is carried out by external assessors. The research team provides a one-day intensive education program consisting of survey procedures, safety and infection education, and an item-by-item explanation. The education session is followed by a Q&A session and three mandatory practice sessions with mock cases of older adults. All outcome data are collected using a tablet PC in which survey instruments have been embedded as a weblink and/or smartphone application. Prior to data collection, all external assessors are obliged to sign to an agreement form for confidentiality concerning information about the participants obtained during the survey.

The procedure for data collection is as follows. Upon enrollment into this intervention study, the recruiting researcher schedules each participant to meet with an external assessor who will visit the participant’s house (or any place that the participant feels comfortable) to conduct the baseline survey. Except for unavoidable circumstances (e.g., resignation of assessor, scheduling issues, etc.), the same assessor is assigned to the participant for the follow-up surveys at Week 13. The contents of the data collected at each time point are described in Table 2. Communication between the research team and the assessors is facilitated through KakaoTalk for assessment scheduling and real-time technical support.

As the intervention is being conducted during the COVID-19 pandemic, special precautions are to be taken during face-to-face interviews. All assessors are given an infection prevention protocol: 1) ensuring the use of facial masks and hand sanitizers for both assessor and participant; 2) daily personal screening for any symptoms; 3) screening at the participant’s doorstep before entry for COVID-19 symptoms, a history of foreign travel within the past 14 days, or visits to a site of mass infection; 4) taking the temperature of participants and nearby family members; 5) sterilizing any equipment used during the interview before moving to the next household. Assessors are required to submit temperature logs for themselves and all participants that they interview.

### Data analysis plan

#### Effect evaluation

Descriptive statistics will be used to analyze data concerning the participants’ demographic and baseline characteristics. Baseline differences between the intervention and control group will be analyzed using an independent sample t-test or the Mann–Whitney U-test for continuous data and the Chi-square test for categorical data. The primary analysis is an evaluation of the interventional effect of the HWePS program in the intervention area as compared to the control area. We will apply mixed effects models and generalized estimating equations adjusting for sample characteristics. In case there are significant baseline differences between groups, propensity score matching methods will be used. Subgroup analyses will be performed for sociodemographic factors (e.g., age, sex, income, etc.) and health status (e.g., frailty), and two paired t-tests will be applied for those variables collected only in the intervention group. All statistical analyses will be carried out using SAS 9.4 (Cary, NC, USA) and p-values with α ≤ 0.05 will be considered significant.

Effect analysis will be conducted based on the intention-to-treat (ITT) principle, in which all participants who participated in the HWePS study will be included regardless of intervention dose or protocol adherence. Imputation methods will be implemented to handle missing data to enable ITT analysis. Sensitivity analyses will be conducted to assess the effects of inclusion in various groups within the samples.

#### Process analysis

Quantitative data will be analyzed using simple descriptive analyses using SAS 9.4 (Cary, NC, USA), including the logs from the HWePS ICT system, the HWePS app, and the Walk-On app (a community walking tracking app) in order to monitor delivery of the intervention. We will also analyze the standardized-questionnaire item responses from participants and lay-leaders, participants’ attendance documents, participants’ self-practice records, etc.

In addition, qualitative data will be analyzed using thematic analysis in MAXQDA qualitative software. These data include the semi-structured questionnaire responses from lay-leaders/multidisciplinary teams, focus group interviews with multidisciplinary teams/lay-leaders/participants, case studies from multidisciplinary team meetings, lay-leader documents, meeting/event reports, etc.

### Ethics and dissemination

This study was approved by the institutional review board (IRB) of human subjects at Seoul National University (SNU IRB 2011/002–016). No critical modifications have been made to the protocol thus far and any changes in the future will be reported to the relevant parties. All data including personal information is saved on a password-protected computer within a locked laboratory, and only the PI and those with permission from the PI will receive access to the final dataset. None of the investigators have financial or competing interests.

The HWePS model is being tested within the context of designing a community care model in preparation for a super-aged society. From the initiation of the study, the contents of the intervention have been designed with community-level dissemination of the model in mind. input from various stakeholders has been sought in the design and implementation process of the intervention. Jungnang-gu and Seoul are planning administrative and educational activities to disseminate the HWePS model, either in part or in whole, into other areas in the district (*gu*) and the city, along with the various protocols/materials/resources developed, if the intervention is effective. Scientific dissemination of the trial results will include presentations at academic conferences, publishing in peer-reviewed journals, and reporting to a trail registry. The results will be reported regardless of effect size or direction. Authorship eligibility will follow the ICMJE guidelines, and we have no plan to hire professional writers.

### Trial status

The study has been rolled out to the intervention and control communities, and data collection is ongoing at the submission of this manuscript. Data analysis has not yet started.

## Discussion

While healthy aging for all is widely accepted public health agenda, few multi-faceted, multi-level health equity interventions promoting health and wellness exist, especially ones targeting relatively healthy older adults residing in an urban, low-income community in a non-Western country. In Korea, public health centers typically offer provider-driven, individual-level, health behavior monitoring or modification programs for people who are willing to participate [60]. Current visiting health services mainly target low-income people and prioritize those with pre-existing serious health conditions requiring immediate, intense attention with limited resources [61]. It is also challenging to organize community-wide activities to address middle- and/or upper-stream social determinants of health in order to reduce inequality in current public health practices [62].

We designed the HWePS to develop and rigorously test the effectiveness of an intervention that simultaneously engages older people and neighbors (as CLHLs) and enlists community-level resources and strengths to promote the health and wellness of older people in a low-income urban community during COVID-19. If effective, the intervention, as a preventive, integrated community care model, may add great value to current efforts to reduce health disparities among urban, lower-income older populations with double (age- and income-related) risks for inequality. First, instead of a professional-driven model, the intervention is an older person-led, community-engaged, professional-support model. As a theory-driven, evidence-based public health model, the HWePS will address health inequality by empowering older adults to recognize that they are responsible for their own health, understand their own health status and risks using a decision-support tool, develop with a nurse coach their own plan for self-care skill-building, and practice it daily with peer support enhanced by ICT tools. Second, the intervention educates and empowers participating CHNs for the “visiting community service center service” program in Seoul [16], and designs and demonstrates new roles for the CHNs, whose current roles are mainly as direct care providers. In the HWePS program, these nurses act as leaders of a multidisciplinary care team, coaches for empowering older people, and mobilizers of community resources and strengths, helping to increase community capacity to build healthy living conditions in collaboration with various stakeholders in the community as well as the multidisciplinary care team. The required preparation, resources, and also challenges for the transformation of CHNs’ roles can be identified and shared with various stakeholders, including CHNs themselves and also administrators and policymakers at various levels involved in this intervention project and more broadly.

Third, peer support is a key mechanism to promote health equity for the community [63] that the HWePS adopted based on existing evidence and input from the various stakeholders in the community during the intensive intervention development process. If effective, the intervention will show that informed, motivated, trained community lay health leaders (CLHLs) can be key players in operating the intervention as close partners with of older community residents (participants) and also as catalysts who play a critical role in the community accepting and embracing the newly developed intervention program as its own. Fourth, we expect ICT tools, digital technologies including the HWePS app and SMS, to act as indispensable tools in the success of the intervention program. In particular, we are testing the effects of a tech-human hybrid model, the use of technology with human (peer) support for an older population with relatively low (e-)health literacy; this can guide a new program of research on designing more effective digital health and wellness programs to build citizen-centered, age-friendly, connected, healthy and caring communities.

There are several expected challenges. One major challenge is the ongoing COVID-19 pandemic; public health measures to stop the infection’s spread could negatively influence various parts of the intervention implementation process (e.g., participation and retention rates) [64], which in turn may impact the results of the study. The COVID-19 pandemic is an unavoidable external threat but one that, in fact, justifies the need for this health equity intervention. To overcome this challenge, rather than the in-person large-group gatherings traditional interventions use, we have developed and implemented alternative-format meetings (e.g., one-to-one meetings, small-group meetings, remote meetings, etc.) within and between older participants, peers, and professional care teams. In addition, various ICT tools have been actively adopted, including the HWePS app, SMS, phone, and virtual meeting services (e.g., Zoom); this is possible as 5G and LTE network service is readily available everywhere across Seoul, including in our intervention community.

Another challenge is that the multi-channel heath information delivery service is designed to be an essential communication channel between the community and the wellness center during COVID-19. However, the basic unidirectional communication approach with limited feedback channels may reduce the intervention effects. While the components and duration of the 12-week (self-)care management intervention are based on existing evidence from successful intervention studies, they may not be enough to motivate older adults to continue healthy lifestyles for prolonged periods. A “companion” program has been organized at the wellness center and is designed as a self-help club in the community run by older people who have completed the 12-week intervention along with support from CLHLs. It is beyond the scope of this intervention study, but the research team plans to assess the effects of the companion program. There are also limited resources, time, and political/administrative networks and support for implementing the community-wide healthy aging initiative in ways that could lead to more meaningful changes. For example, while participants have expressed income-based health-related social needs, there are limited income-earning opportunities available in the community for older people; changing upstream social/structural determinants of health inequality is hard to address in this equity intervention study. If the intervention is effective, we will help facilitate the public health center’s continuation of the HWePS program in the intervention area, promoting it as a signature program for improving the living conditions of the area, and support the program’s dissemination to other areas of Jungnang-gu and beyond; we will also encourage the Community Residence Council to enact bylaws making a new health committee under its purview.

## Data Availability

The datasets generated and/or analyzed during the current study are not publicly available due to the policy of the SNU IRB, which does not allow the opening and sharing of research data with any third party, but are available from the corresponding author upon reasonable request.

## Abbreviations

6SQuID: Six Steps for Quality Intervention Development
BARHII: Bay Area Region Health Inequity Initiative
CCM: Chronic care model
CHN: Community health nurse
CLHL: Community lay health leader
CPAs: Community participatory activities
CSDH: Commission on Social Determinants of Health
COVID-19: Coronavirus disease 2019
CUSR: Check-up self-report
ECCM: Expanded chronic care model
HL: Health literacy
HwEPS: Health and Wellness Program for Seniors
ICT: Information and communications technology
IDP: Intervention development process
ITT: Intention to treat
MCC: Multidisciplinary care conference
SMS: Short message service
SPEC: System for Person-centered Elderly Care
TREND: Transparent Reporting of Evaluations with Nonrandomized Designs
